# Supplementary figures and images for: Pan-Genome Analysis of Wolbachia, Endosymbiont of Diaphorina citri, Reveals Independent Origin in Asia and North America
Source: Int J Mol Sci. 2024 Apr 29;25(9):4851. doi: 10.3390/ijms25094851 (PMC11084931; doi:10.3390/ijms25094851)

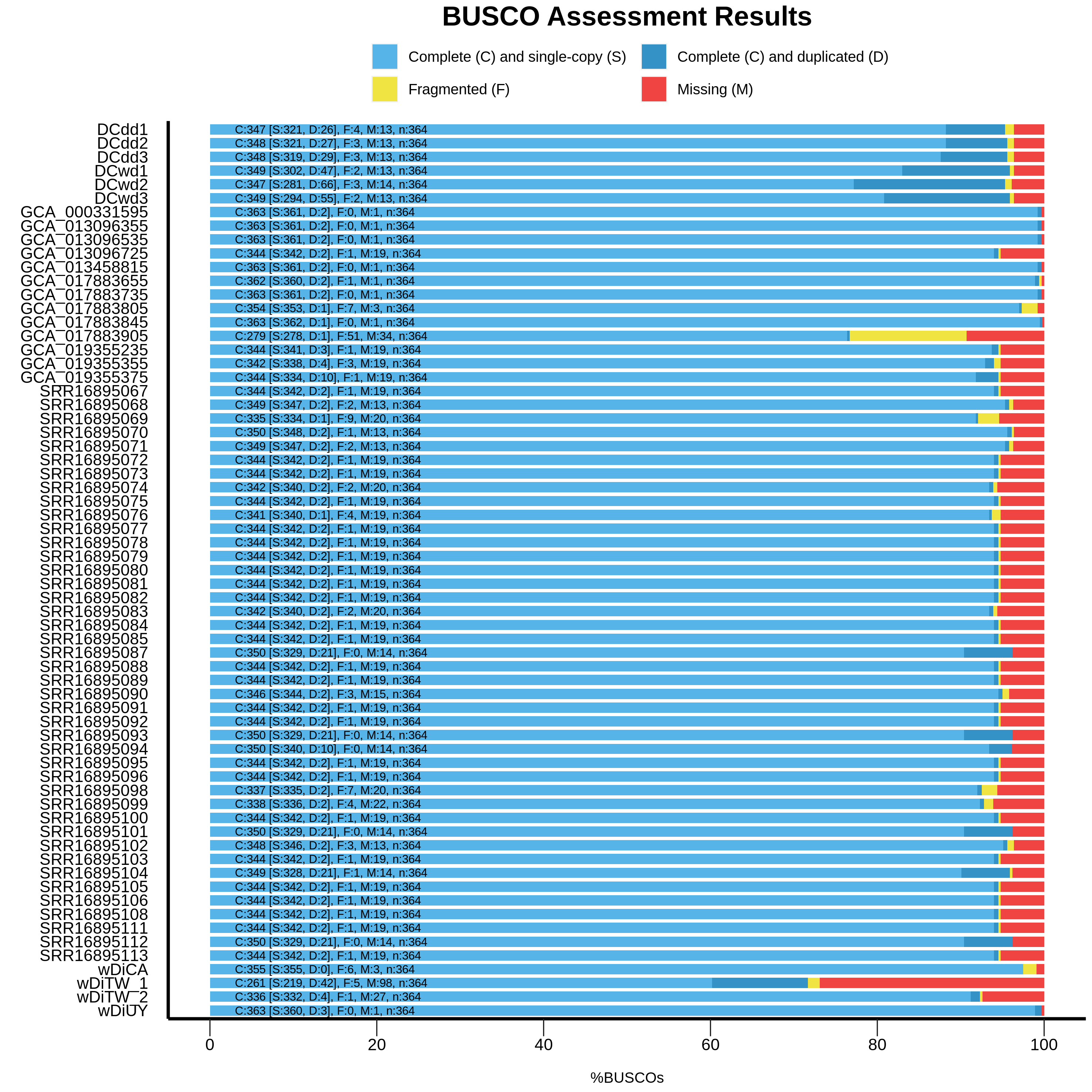

Supplement: Supplementary file 1 [file ijms-25-04851-s001.zip › Figure S1.png]

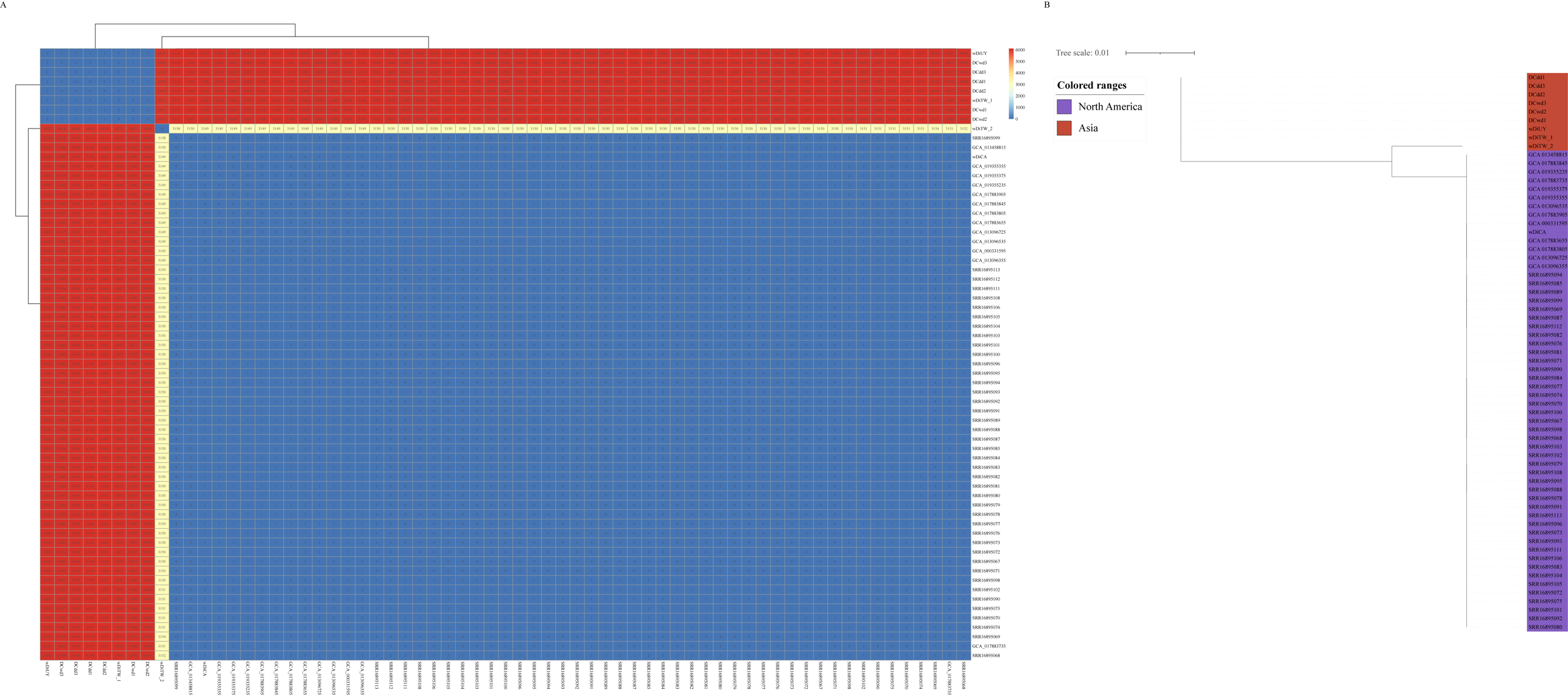

Supplement: Supplementary file 1 [file ijms-25-04851-s001.zip › Figure S2.tif]
